# Effects of Social Participation by Middle-Aged and Elderly Residents on the Utilization of Medical Services: Evidence From China

**DOI:** 10.3389/fpubh.2022.824514

**Published:** 2022-07-07

**Authors:** Tai-Yi Liu, De-Chao Qiu, Ting Chen

**Affiliations:** ^1^School of Public Health, Hubei Provincial Key Laboratory of Occupational Hazard Identification and Control, Wuhan University of Science and Technology, Wuhan, China; ^2^Jintang First People's Hospital, West China Hospital Sichuan University, Jingtang, China

**Keywords:** social participation, utilization of medical services, propensity score matching, elderly residents, middle-aged residents

## Abstract

**Objectives:**

Aim to evaluate the effect of social participation on utilization of medical services among middle-aged and elderly residents in China.

**Methods:**

We used data from the 2018 wave of the China Health and Retirement Longitudinal Study. Social participation is classified into three types. Furthermore, to control for confounding factors, our study computed propensity score matching (PSM) to evaluate the effect of social participation on the utilization of medical services.

**Result:**

The result of PSM indicates that social participation significantly positively affects the utilization of outpatient services, the average treatment effect on the treated (ATT = 0.038^***^) and the utilization of inpatient services (ATT = 0.015^**^) by middle-aged and elderly residents. Furthermore, the utilization of outpatient health care services was significantly positively associated with leisure activities (ATT = 0.035^***^), social activities to help others (ATT = 0.031^***^), and learning activities to gain new knowledge (ATT = 0.034^***^) among middle-aged and elderly residents. The utilization of inpatient health care was significantly positively associated with leisure activities (ATT = 0.015^***^) but had no significant association with social deeds that help others and increased new knowledge among middle-aged and elderly residents.

**Conclusion:**

Thus, social participation significantly positively affects healthcare utilization by middle-aged and elderly residents. Hence, the government and society should provide more conveniences and promote social participation among middle-aged and elderly residents.

## Introduction

Population aging is a global problem, and China has the most rapidly aging population in the world (1). With the aging of the people in China, diseases among older adults, such as chronic non-communicable diseases and mental health disorders, have gained increasing attention (2), resulting in an emerging demand for health services by middle-aged and elderly residents (3).

The Andersen Health Services Utilization Initial Model, which includes predisposing, enabling, and need factors, is one of the most widespread research frameworks to indicate the influence of a healthcare utilization factor (4). Among the middle-aged and elderly residents, physical, and mental health had a negative connection with the utilization of medical services (5, 6), and social variables, such as regular involvement in activities of clubs, societies, and religious organizations, increase the odds of health service utilization (7).

Social participation was defined as participation in social activities, including engagement as volunteers and social groups interacting with the community (8). It is common knowledge that middle-aged and elderly residents have a relatively lower social participation rate. In addition, social participation is positively associated with improved overall health, which increases with age (9, 10). Besides providing better health, social participation improves the social bonds of middle-aged and elderly residents, and older people stay active by participating in voluntary work (11).

Although social participation is meaningful, few studies have examined its influence on the utilization of health services. Research reveals that the association between social participation and health services is complex. The results were affected by other factors, including health factors (12, 13). One study that used a nationally representative sample of older adults found that people with high social participation were significantly less likely to utilize health care services (visit their family doctor and district nurse last month). When controlling for demographic factors, physical and mental health, and physical activity, the longitudinal data indicated that social participation was positively associated with contacting home help services (13). Another researcher noted that social participation was significantly connected with low inpatient visits by controlling initial health variables (12).

The association between social participation and healthcare utilization due to confounding factors is unclear. Therefore, this study used the propensity score method (PSM) to control for confounding factors and explore the effect of social participation on the utilization of health care services among middle-aged and elderly residents. PSM is a statistical technique that can effectively reduce selection bias due to confounding variables between two different groups, such as the number of social participants and unsociable participants among middle-aged and elderly residents (14). PSM is widely used in social participation or medical service utilization. Yean Wang divided the middle-aged and elderly into two groups: internet users and non-internet users, and used the PSM method to analyze the connection between Internet use and levels of depression. The results show that older adults who reported internet use have lower depression levels than did those who did not use the internet, with adjustments made for Confounding factors (15). Jong-Yi Wang found that mortality and total medical expenditures per capita were significantly higher in psychiatric patients than in non-psychiatric patients by 1:1 dual propensity score matching (16).

## Methods

### Data Sources and Sample Selection

The data from the 2018 wave of the China Health and Retirement Longitudinal Study (CHARLS) was a nationally representative longitudinal study conducted by the China Center for Economic Research (CCER), Peking University (17). The baseline survey of CHARLS was performed in 2011, and samples were surveyed every 2 years. Four national study waves of national study until 2018, analyzing 17,543 Chinese residents over 45 years old. Participants were selected from 450 villages and urban communities of 28 provinces in China, through probability-proportional-to-size sampling, using a multistage stratified sampling technique. They were interviewed through a high-quality questionnaire, which required detailed information about demographic backgrounds, health care and status, cognition, depression, work retirement, and property ownership (18).

The Biomedical Ethics Review Committee approved the CHARLS Study at Peking University in January 2011. All enrollees were notified, and informed consent was obtained before the interviews. We applied to the CHARLS team for data for this study and received anonymous enrollees (19).

**Figure 1:**
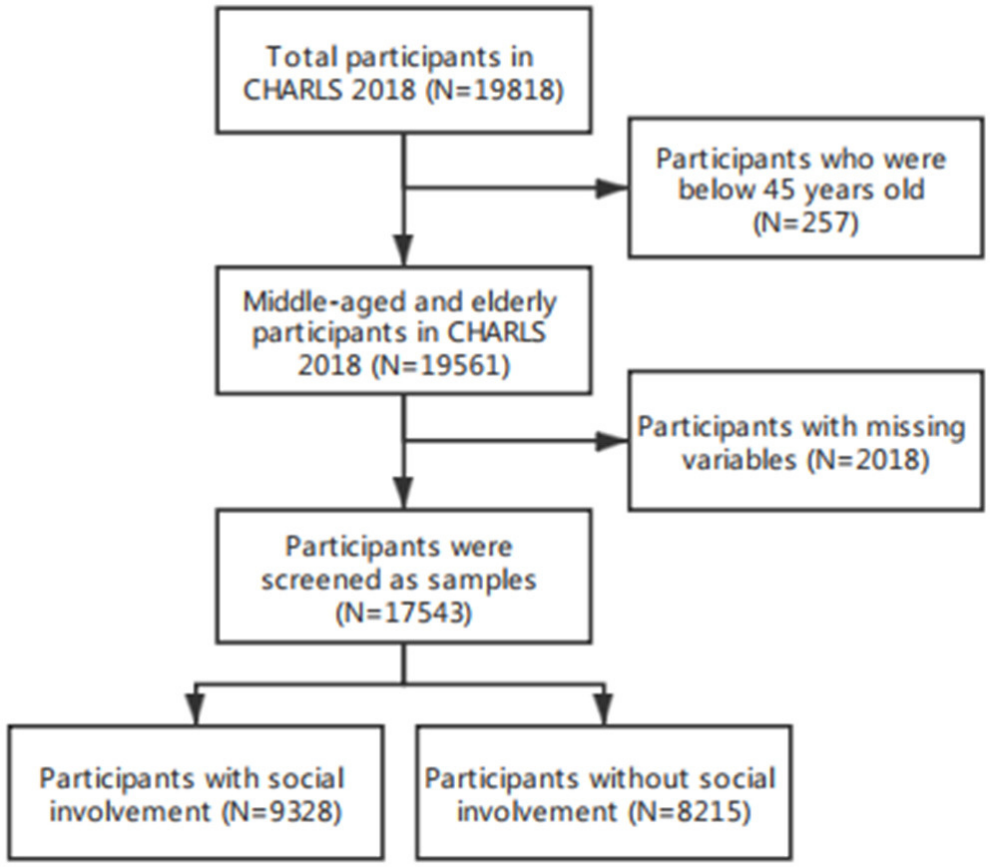
Flowchart of the observation's selection and classification; CHARLS, (China Health Retirement Longitudinal Study).

To comply with the research purpose, the invalid samples from 2018 comprised 257 participants aged 45 years, and 2018 participants with missing variables were eliminated. The final sample consisted of 17,543 enrollees, including 9,328 enrollees who participated socially and 8,215 participants who did not. The effective sample rate was 88.5% (Figure 1).

### Measurements

#### Social Participation

To measure current social participation, we asked participants 10 multiple-choice questions about their involvement in the following activities, in the previous month: (1) interacting with friends; (2) playing Mahjong, playing chess, playing cards, or going to a community club; (3) providing help to family, friends, or neighbors who do not live with you; (4) went to the sport, social, or other kind of the club; (5) took part in a community-related organization; (6) voluntary or charity work; (7) cared for a sick or disabled adult who does not live with you; (8) attended an educational or training course; (9) stock investment; and (10) using the internet. If respondents answered at least one “yes” to the above questions, they were defined as “social participants.” The other respondents were defined as unsociable participants (20).

We classified social activities into three types to explore the associations between various categories of social participation and the utilization of medical services. The labels of training, 1, 2, 4, and 5, were regarded as leisure activities; the classifications of action, which were 3, 6, and 7, were considered as social activities to help others; the categorization of training, consisting of 8, 9, and 10, were regarded as learning activities to acquire new knowledge (21). Among 17,543 middle-aged and elderly residents, 7,797 (44.5%) enrollees engaged in leisure activities, 2,831(16.1%) partook in social activities to help others, and 2,286 (13.0%) joined learning activities for new knowledge.

#### Utilization of Medicine Services

This research included two types of services for medicine utilization: (1) outpatient visits: we asked participants whether they had visited a hospital, public health center, clinic, or were seen by health workers or doctors for outpatient care, last month; (2) inpatient visits: participants who had received inpatient care in the past year (22). The variables of outpatient and inpatient utilization was the classification variable (1 = yes, 0 = no).

#### Covariables

According to the Andersen Health Services Utilization Initial Model framework, we classified the covariates that were comprehensively arranged and screened from the CHARLS questionnaire into three elements with some effect on the utilization of health services (23).

The parts included:(1) Propensity characteristics; age, gender, education level (no formal education, primary school, middle school, high school, and above), marriage (married and currently living with a spouse or others), household register (urban, rural), number of family members: (2) Capacity resources; income (household income in the past year), insurance (had at least one type of insurance or others), receive from child (financial support received from children, last year), provide to children (financial aid provided to children, last year): (3) Health needs; smoke (smoking currently, never smoked, stopped smoking), drink (drink alcohol now, never drank, stooped drinking), health status (perfect, good, poor, very poor), chronic disease (had at least one type of chronic illness or others), the activities of daily living (ADL) (the capacity of daily living activity, had 20 questions and a total of 80 points. The higher the score, the lower the ADL score). Covariables, including “income,” “receive from the child,” and “provide to the child” were natural logarithm transformed, as the original data were skewed to the right.

### Statistical Analysis

The *t*-test and Pearson chi-square test were used for the social participation survey to analyse the differences in demographic characteristics between the social and unsociable participation groups.

The primary statistical analysis method used was the propensity score matching (PSM) model computed through psmatch2 of Stata 15.0. The PSM model was a non-parametric statistical method to reduce the selective bias between treatment and control units (18, 24). The confounding factors were balanced, based on the propensity score by matching two groups, reflecting the selective probability of receiving treatment. The average treatment effect on the treated (ATT) was estimated after the PSM method reduced the influence of confounding variables in matched groups, which was the mean of the individual causal effect in the treated unit (25).

PSM analysis was classified into three steps. The first was calculating the propensity score according to the logistic model, including the abovementioned covariates. Second, the treatment and control groups were matched based on the propensity score, where matching methods varied in PSM, including the *k*-nearest neighbor matching method, radius matching method, and kernel matching method (15). Third, the ATT was evaluated. If the value of ATT is positive and statistically significant, it represents that social participation has increased the healthcare utilization rate. Suppose the value of ATT is negative and statistically significant. In that case, it means that social participation has decreased the health care utilization rate, such as ATT of social participation was 0.061 to inpatient visits, showing that the social participation had increased the inpatient visit rate within 1 year by approximately six percent points (26). The kernel matching method at a bandwidth of 0.06 was used, as frequently computed in the literature (27, 28). The *k*-nearest neighbor matching method (one to four), and the radius matching method (Caliper = 0.03) confirmed the robustness of the results.

The PSM method resulted in erroneous standard errors owing to the high correlation between matched samples and the error between the estimated propensity score and the actual value. The current approach is to substitute the standard empirical mistake with the empirical standard error. Our research used the bootstrap method to obtain the empirical standard error (15).

### Patient and Public Involvement

The data used in this study were derived from the CHARLS database, so patients and the public were not involved in the design or conduct of this research.

## Results

### Characteristics of Study Samples

Samples regarding social activity were selected in two groups and descriptive analyses were conducted for the differences between the groups (*t*-test was computed for continuous variables, and chi-square test was computed for classification variables). For outcome variables, social participants were more likely to visit outpatients (*p* < 0.001), and unsociable participants were likelier to visit inpatients (*p* < 0.001). There were statistically significant social participation differences between the two groups of middle-aged and elderly residents on 11 covariates: middle-aged and older residents who were younger (*p* < 0.001), had a higher education level (*p* < 0.001), had a spouse (*p* < 0.001), provided for children more (*p* < 0.001), had higher income (*p* < 0.001), had insurance (*p* < 0.001), smoked (*p* < 0.001), drank (*p* < 0.001), had better health (*p* < 0.001), had lower ADL (*p* < 0.001), were urban residents (*p* < 0.001), had a higher incidence of social participation. This finding suggests that the effect of social participation on the utilization of medical services is complicated to calculate (Table 1).

**Table 1:** Characteristics of social and unsociable groups before matching.

**Variables**	**Social participants**	**Unsociable participants**	***p*-value**
	**(*N* = 9,328)**	**(*N* = 8,215)**	
**Outcome variables**			
Outpatient visit (last month)			<0.001
Yes	1,683 (18.04)	1,242 (15.12)	
No	7,645 (81.96)	6,973 (84.88)	
Inpatient visit (last year)			<0.001
Yes	1,480 (15.87)	1,496 (18.21)	
No	7,848 (84.13)	6,719 (81.79)	
**Covariate**			
Gender			
Male	4,481 (48.04)	3,865 (47.05)	0.190
Female	4,847 (51.96)	4,350 (52.95)	
Age	60.74 ± 0.10	63.78 ± 0.11	<0.001
Education level			<0.001
No formal education	1,587 (17.01)	2,415 (29.40)	
Primary school	3,888 (41.68)	3,753 (45.68)	
Middle school	2,335 (25.03)	1,485 (18.08)	
High school and above	1,518 (16.27)	562 (6.84)	
Marriage			<0.001
Having spouse	8,083 (86.49)	6,941 (84.49)	
No spouse	1,245 (13.35)	1,274 (15.51)	
Receive form child	5.98 ± 3.66	5.99 ± 3.49	0.836
Provide to child	2.85 ± 3.64	1.84 ± 3.09	<0.001
Income	9.18 ± 2.58	8.59 ± 2.66	<0.001
Health insurance			<0.001
Yes	9,099 (97.55)	7,931 (96.54)	
No	299 (2.45)	284 (3.46)	
Drink			<0.001
Yes	3,595 (38.54)	2,336 (28.44)	
No	5,733 (61.46)	5,879 (71.56)	
Smoke			<0.001
Yes	2,670 (28.62)	2,077 (25.28)	
No	6,658 (71.38)	6,138 (74.72)	
Number of family members	2.66 ± 1.15	2.68 ± 1.22	0.471
Health status			<0.001
Very good	1,193 (12.79)	842 (10.25)	
Good	1,335 (14.31)	872 (10.61)	
Pair	4,629 (49.62)	3,828 (46.48)	
Poor	1,684 (18.05)	2,002 (24.37)	
Very poor	487 (5.22)	681 (8.29)	
Chronic disease			0.119
Yes	7,451 (78.88)	6,639 (80.82)	
No	1,877 (20.12)	1,576 (19.18)	
ADL	20.89 ± 8.89	25.13 ± 12.55	<0.001
Household register			<0.001
Urban	4,041 (43.32)	2,695 (32.81)	
Rural	5,287 (56.68)	5,520 (67.19)	

*t-test was computed for continuous variables, and chi-square test was computed for classification variables*.

*ADL, activities of daily living*.

### Association Between Social Participation and the Utilization of Medicine Services

Table 2 shows results for the overall balance *t*-test for 15 covariates. After kernel matching the propensity score, the final sample size in this analysis was 9,325 social participation enrollees and 8,199 unsociable participation enrollees. The bias of overall covariates was lower than 5% (mean bias = 1.7%, median bias = 1.4%) and no longer significant between the two groups (*p* = 0.294 > 0.05); the covariates of the two groups were significantly balanced, and PSM improved the comparability of social participation enrollees and unsociable participation enrollees.

**Table 2:** Overall balance *t*-test and the average treatment effect on the treated of social participation.

	**Overall balance**					**Match result**							
						**Outpatient visits**				**Inpatient visits**			
						**ATT**	**Bootstrap result**			**ATT**	**Bootstrap result**		
	**Pseudo**	**LR**	***p*-**	**Mean**	**Median**		**Bootstrap**	***z***	***p*-**		**Bootstrap**	***z***	***p*-**
	**R^**2**^**	**CHI^**2**^**	**value**	**bias**	**bias**		**SE**		**value**		**SE**		**value**
Unmatched	0.067	1,626.06	<0.001	16.9	20.7	0.029^***^				−0.023^***^			
Matched	0.001	17.43	0.294	1.7	1.4	0.038^***^	0.006	6.36	<0.001	0.015^**^	0.005	2.82	0.005

*ATT, the average treatment effect on the treated*.

^*^p < 0.10,

** *p < 0.05*,

*** *p < 0.01*.

The ATT of the raw sample and the sample after kernel matching can be estimated. The estimates reveal that middle-aged and elderly residents who participated socially in the last month had significantly higher outpatient visit rates than those who had not attended socially in the previous month in both the unmatched sample and the kernel matched sample (ATT = 0.029^***^; 0.038^***^). Furthermore, the inpatient visits showed a higher rate in middle-aged and elderly residents who did not participate socially in the last month than those who had, before kernel matching (ATT = −0.023^***^). However, after kernel matching, the middle-aged and elderly residents who participated socially in the last month showed a higher inpatient visit rate than those enrollees who had not (ATT = 0.015^**^) (Table 2).

### Effects of Three Types of Social Participation on the Utilization of Medical Services

Table 3 describes the impact of diverse kinds of social participation on the utilization of medical services. The treatment group and control group's overall balance for each type displayed excellent results for covariate balances after kernel matching. The mean and median biases were jointly lower than 5% (mean bias = 1.1%; 1.9%; 1.7%, median bias = 0.7%; 1.5%; 1.1%), and no longer significant between the two groups in the three types of social participation (*p* = 0.910, 0.979, and 0.979, 0.05).

**Table 3:** Overall balance *t*-test and propensity score matched results and effect of each type of social participation.

**Variables**	**Overall balance**					**Match result**							
						**Outpatient visits**				**Inpatient visits**			
						**ATT**	**Bootstrap result**			**ATT**	**Bootstrap result**		
	**Pseudo**	**LR**	***p*-**	**Mean**	**Median**		**Bootstrap**	***z***	***p*-**		**Bootstrap**	***z***	***p*-**
	**R^**2**^**	**CHI^**2**^**	**value**	**bias**	**bias**		**SE**		**value**		**SE**		**value**
**Leisure activities**													
Unmatched	0.041	993.47	0.001	12.3	16.3	0.027^***^				−0.012^**^			
Matched	0.001	8.32	0.910	1.1	0.7	0.035^***^	0.006	6.29	<0.001	0.015^***^	0.005	2.90	0.004
**Social activities helping others**													
Unmatched	0.052	811.00	0.001	17.8	13.3	0.025^***^				−0.033^***^			
Matched	0.001	6.05	0.979	1.9	1.5	0.031^***^	0.008	4.06	<0.001	0.002	0.007	0.30	0.765
**Learning activities**													
Unmatched	0.266	3,611.83	0.001	42.1	40.6	0.021^**^				−0.054^***^			
Matched	0.001	6.06	0.979	1.7	1.1	0.034^***^	0.01	3.26	<0.001	0.003	0.009	0.36	0.719

*ATT, the average treatment effect on the treated*.

^*^p < 0.10,

** *p < 0.05*,

*** *p < 0.001*.

Among middle-aged and elderly residents, social participation (leisure activities, social activities helping others, and learning activities) significantly improved the outpatient visit rate in both raw samples and matched samples after kernel matching (ATT = 0.035^***^; 0.031^***^; 0.034^***^). For inpatient visits, all types of social participation had a significantly negative role before matching (ATT = −0.012^**^; −0.033^***^; −0.054^***^), and after kernel matching, the results changed. The inpatient visit rates were positively and significantly different between the treatment unit and control unit for leisure activities (ATT = 0.015^***^), and there was no significant difference between the two groups in the two other types of social participation (ATT = 0.002, 0.003). Table 4 showed the combined effect of different types of social participation on utilization of medical services. Compared with those who did not participate in any social participation, participants who attended one type of social participation (any one of leisure activities, social activities helping others, or learning activities) had a significantly higher outpatient visit rate and inpatient visit rate after kernel matching (ATT = 0.029^***^, ATT = 0.014^**^). The effect was even more significant in the participants who attended two or three types of social participation (ATT = 0.057^***^, ATT = 0.017^**^).

**Table 4:** Overall balance *t*-test and propensity score matched results and combined effect of each type of social participation.

**Variables**	**Overall balance**					**Match result**							
						**Outpatient visits**				**Inpatient visits**			
						**ATT**	**Bootstrap result**			**ATT**	**Bootstrap result**		
	**Pseudo**	**LR**	***p*-**	**Mean**	**Median**		**Bootstrap**	***z***	***p*-**		**Bootstrap**	***z***	***p*-**
	**R^**2**^**	**CHI^**2**^**	**value**	**bias**	**bias**		**SE**		**value**		**SE**		**value**
**Social participation (Ref: none)**													
**One type**													
Unmatched	0.032	632.92	0.001	10.7	13.2	0.022^***^				−0.013^**^			
Matched	0.001	9.44	0.853	1.1	0.8	0.029^***^	0.006	4.71	<0.001	0.014^**^	0.006	2.41	0.016
**Two or three types**													
Unmatched	0.188	2,479.74	0.001	32.3	36.5	0.043^***^				−0.047^***^			
Matched	0.001	7.81	0.931	1.7	1.4	0.057^***^	0.009	6.00	<0.001	0.017^**^	0.008	2.17	0.030

*ATT, the average treatment effect on the treated*.

^*^p < 0.10,

** *p < 0.05*,

*** *p < 0.001*.

### Robustness Check

Our research compared the results of the kernel matching method with radius and *k*-nearest neighbor matching methods (one to four) and Logistic regression to explore the robustness of the kernel matching method in propensity score matching. Table 5 indicates that the significance of the average treatment effect for the matching methods was the same. The propensity score-matching results were robust.

**Table 5:** The average treatment effect of propensity score matching among three matching methods and logistic regression.

**Variables**	**Outpatient visits**				**Inpatient visits**			
	**Model 1**	**Model 2**	**Model 3**	**Model 4**	**Model 1**	**Model 2**	**Model 3**	**Model 4**
	**ATT**	**ATT**	**ATT**	**OR**	**ATT**	**ATT**	**ATT**	**OR**
Social participation	0.038^***^	0.038^***^	0.039^***^	1.356^***^	0.015^**^	0.016^**^	0.015^**^	1.121^**^
(1) Leisure activities	0.035^***^	0.035^***^	0.037^***^	1.314^***^	0.015^**^	0.015^**^	0.012^**^	1.140^***^
(2) Social activities helping others	0.031^***^	0.031^***^	0.037^***^	1.266^***^	0.002	0.004	0.008	1.046
(3) Learning activities	0.034^***^	0.034^***^	0.030^***^	1.283^***^	0.003	0.004	0.005	1.011

*ATT, the average treatment effect on the treated; Model 1: Kernel matching method in PSM, Model 2: Radius matching method in PSM, Model 3: k-nearest neighbor matching method in PSM, Model 4: Logistic regression*.

^*^p < 0.10,

** *p < 0.05*,

*** *p < 0.01*.

## Discussion

To the best of our knowledge, this is the first study that used PSM to assess the association between social participation and healthcare utilization. Our research explored the association between social participation and healthcare utilization by PSM among residents aged 45 years and above based on the 2018 CHARLS statistics. By adjustment for 15 confounding variates through PSM, this study demonstrated a significant positive effect of social participation on healthcare service utilization by middle-aged and elderly residents. All the treatment groups; leisure activities, social helping activities, and learning activities, had a higher rate of outpatient visits than their corresponding control groups. Moreover, leisure activities enrollees had a healthy level of inpatient visits in contrast with the team that had not.

In China, there were about 249.49 million residents aged 60 and over at the end of 2018 (29). Severe population aging is a burden on health care utilization; health is a significant relevant intermediary element; and unmet health care needs is deleterious for older adults (30, 31). The association between health care utilization and unmet health care needs was remarkably negative, and the promoted utilization of medical services met health care needs (32).

We found that older men had less social participation than younger men, and the rate of residents over 45 years who participated socially was 53.17%. The incident odds of social participation in prior studies paralleled those in our study. The number of residents participating socially was not high (33, 34). In contrast, the elderly in developed countries usually had high social participation rates (29).

Moreover, regardless of outpatient and inpatient services, residents with greater social participation, utilized health care services more. There are many possible reasons for this finding. First, social activities improved relationships with relatives and friends, which confirmed the optimism associated with the utilization of medical services and preventive health care (35, 36). Improving relationships with friends and relatives may reduce the psychological pressure and financial support of middle-aged and older adults facing medical treatment. Second, previous research shows that socially isolated older adults used health care services less, and there was a significant association between loneliness and lower odds of physician visits, social isolation and loneliness will lead to the weakening of mobility and the obstruction of social communication in middle-aged and older adults (37, 38). Social participation reduces social isolation and helps the elderly to integrate socially. Furthermore, our findings revealed that social participation had a negative connection to the incidence of inpatient visits before matching, similar to previous research that indicates that higher social participation meant lower odds of inpatient visits (12). However, the result changed after matching the factors, possibly because of the covariates for health needs, which most significantly affected the utilization of medical services in the Andersen model.

In Previous researches, the relationship between social participation and medical service utilization was unclear. It was often mixed with other factors, such as health factors. Since health factors were a relatively crucial confounding factor, the opposite result may be obtained after the balance of health factors (12, 13). The same pattern was present in our study, social participants had higher health status, and those with solid health had lower healthcare utilization, utilization of medical services will increase with the balance of health-related variables (23, 39). If the elderly healthy balance to the same level to participate in social activities of the high degree of the elderly, the possibility of medical service utilization will increase. The reasons for the changes in the results before and after the propensity score matching was balancing confounding factors and reducing the influence of confounding factors. The PSM method eliminated the effect of covariates, including health need factors and evaluated the natural result of the connection between social participation and health care utilization.

Our study showed a significantly positive association between diverse types of social participation and outpatient service utilization, and that social participation for leisure, affects inpatient service utilization. Various kinds of social participation revealed diverse characteristics and functions of health care utilization. Leisure activities, such as interacting with friends and playing Mahjong, could enhance relatives' and friends' relationships and relax older adults (40, 41). Social activities to help others, such as voluntary or charity work, generates social integration and fulfillment among older people (21, 42). Learning activities, such as internet use, improved communication and social connections across geographic distances at comparably low consumption (43), and could provide new information about medical services for the elderly. Enhancing the social participation of older adults was a critical factor in successful aging that many older adults value. The government and society should increase the convenience of and promote social participation by providing public spaces to encourage increased social participation in middle-aged and elderly residents.

There are some limitations to our study. First, the 11 covariates in this study may not cover all essential confounding factors for medical service utilization and social participation among middle-aged and elderly residents. Unobservable covariates increase selection bias, influencing PSM results between the treatment and control groups. Second, our study has one indicator that determines social participation in the last month; instead the frequency of current social participation could be examined. The study may ignore the effects of different degrees of social participation on health service utilization. These limitations deserve further scrutiny.

## Conclusions

Our research indicated that residents in middle-age and at a later life stage in China who participated socially, had a significantly higher incidence of utilizing health services than those who did not participate socially. Diverse social activities positively affected outpatient service utilization, and leisure activities were positively associated with inpatient service utilization. Considering our conclusion, the government and society should increase the convenience of and promote social participation by providing public spaces to encourage increased social participation in middle-aged and elderly residents.

## Data Availability Statement

The datasets presented in this study can be found in online repositories. The names of the repository/repositories and accession number(s) can be found below: (http://charls.pku.edu.cn) China Health and Retirement Longitudinal Study.

## Ethics Statement

The studies involving human participants were reviewed and approved by the Biomedical Ethics Review Committee. The patients/participants provided their written informed consent to participate in this study.

## Author Contributions

T-YL, TC, and D-CQ contributed to the development of the research ideas and obtained the dataset. T-YL cleaned the dataset. T-YL and TC performed data analyses and drafted the manuscript. All authors commented on the manuscript. All authors contributed to the article and approved the submitted version.

## Funding

This study was supported by the National Natural Science Foundation of China (Grant Number: 71503090).

## Conflict of Interest

The authors declare that the research was conducted in the absence of any commercial or financial relationships that could be construed as a potential conflict of interest.

## Publisher's Note

All claims expressed in this article are solely those of the authors and do not necessarily represent those of their affiliated organizations, or those of the publisher, the editors and the reviewers. Any product that may be evaluated in this article, or claim that may be made by its manufacturer, is not guaranteed or endorsed by the publisher.
